# The diagnostic accuracy of clinical tests for anterior cruciate ligament tears are comparable but the Lachman test has been previously overestimated: a systematic review and meta-analysis

**DOI:** 10.1007/s00167-022-06898-4

**Published:** 2022-02-12

**Authors:** Pawel A. Sokal, Richard Norris, Thomas W. Maddox, Rachel A. Oldershaw

**Affiliations:** 1 Department of Musculoskeletal and Ageing Science, Institute of Life Course and Medical Sciences, Faculty of Health and Life Sciences, University of Liverpool, William Henry Duncan Building, 6 West Derby Street, Liverpool, L7 8TX UK; 2 Department of Medicine, School of Medicine, University of Liverpool, Liverpool, L69 3GE UK; 3 Department of Trauma and Orthopaedics, Aintree University Hospital, Liverpool University Hospitals NHS Foundation Trust, Lower Lane, Fazakerley, Liverpool, L9 7AL UK; 4 Small Animal Teaching Hospital, Institute of Infection, Veterinary and Ecological Sciences, Faculty of Health and Life Sciences, University of Liverpool, Leahurst Campus, Chester High Road, Neston, CH64 7TE Wirral UK

**Keywords:** ACL, ACL tear, Clinical diagnostic tests, Knee injuries, Sporting injuries, Anterior drawer, Pivot shift, Lever sign, Lachman, Meta-analysis

## Abstract

**Purpose:**

The diagnostic accuracy of clinical tests for anterior cruciate ligament injury has been reported in previous systematic reviews. Numerous studies in these reviews include subjects with additional knee ligament injury, which could affect the sensitivity of the tests. Meta-analyses have also been performed using methods that do not account for the non-independence of sensitivity and specificity, potentially overestimating diagnostic accuracy. The aim of this study was to report the diagnostic accuracy of clinical tests for anterior cruciate ligament tears (partial and complete) without concomitant knee ligament injury.

**Methods:**

A systematic review with meta-analysis was performed according to the PRISMA guidelines. Meta-analyses included studies reporting the specificity and/or sensitivity of tests with or without concomitant meniscal injury. Where possible, pooled diagnostic estimates were calculated with bivariate random-effects modelling to determine the most accurate effect sizes. Diagnostic accuracy values are presented for the anterior drawer, Lachman, Lever sign and pivot shift tests overall and in acute or post-acute presentations.

**Results:**

Pooled estimates using a bivariate model for overall sensitivity and specificity respectively were as follows: anterior drawer test 83% [95% CI, 77–88] and 85% [95% CI, 64–95]; Lachman test 81% [95% CI, 73–87] and 85% [95% CI, 73–92]; pivot shift test 55% [95% CI, 47–62] and 94% [95% CI, 88–97]; Lever sign test 83% [95% CI, 68–92] and 91% [95% CI, 83–95]. For specific presentations, the sensitivity and specificity of the Lachman test, respectively, were: complete tears 68% [95% CI, 54–79] and 79% [95% CI, 51–93]; post-acute injuries 70% [95% CI, 57–80] and 77% [95% CI, 53–91].

**Conclusions:**

The pivot shift and Lever sign were the best tests overall for ruling in or ruling out an anterior cruciate ligament tear, respectively. The diagnostic accuracy of the Lachman test, particularly in post-acute presentations and for complete tears, is lower than previously reported. Further research is required to establish more accurate estimates for the Lachman test in acute presentations and partial ligament tears using bivariate analysis.

**Level of evidence:**

III.

**Supplementary Information:**

The online version contains supplementary material available at 10.1007/s00167-022-06898-4.

## Introduction

Anterior cruciate ligament (ACL) injuries are common with a median annual incidence of 0.03% per person overall and up to 3.7% in professional athletes [52]. Potential consequences of an ACL tear include further knee injury, post-traumatic osteoarthritis, and reduced quality of life [21]; therefore, prompt, accurate diagnosis is important to expedite treatment and mitigate these risks.

ACL tears are diagnosed by combining patient history and physical examination with imaging modalities utilised if required [21]. History elements include a traumatic pivoting mechanism, typically without direct contact to the knee, a ‘popping’ or ‘snapping’ sensation, effusion within 2 h of injury and knee instability [13, 15, 25, 37, 75, 76]. Based on an overview of systematic reviews investigating the diagnostic validity of physical examination tests, the Lachman test is considered to be of high diagnostic value to confirm and exclude an ACL tear, while the pivot shift test may be used to rule in an ACL injury when positive [14]. The Lever sign demonstrates similar diagnostic accuracy to more established tests [2, 59], but these tests have not been compared directly using the same inclusion and exclusion criteria.

Although this synthesis of data represents the best available evidence to guide clinical practice [21], numerous studies in the aforementioned systematic reviews include subjects with additional knee ligament injury [4, 7, 28, 34, 47, 50, 77], which could affect test sensitivity. Since up to half of all patients with an ACL tear sustain a concomitant medial or lateral ligament injury [1, 55, 63], it is important to determine the diagnostic accuracy of ACL tests in the absence of such injury. Furthermore, previous meta-analyses have evaluated studies by methods that do not account for the non-independence of sensitivity and specificity, which are often negatively correlated. More recently, approaches such as bivariate random effects models have been recommended for meta-analysis of diagnostic test accuracy [27, 60].

The purpose of this systematic review with meta-analysis was to provide an updated synthesis of studies reporting the diagnostic accuracy of clinical tests for ACL tears (partial and complete) without concomitant knee ligament injury. Data are presented for the anterior drawer, Lachman, Lever sign and pivot shift tests [24, 42, 46, 73] performed without anaesthesia, in acute and post-acute presentations.

The study hypotheses were that ACL clinical tests will have lower sensitivity in the absence of concomitant ligament injury, and the diagnostic accuracy of the Lachman test will be superior to the Lever sign. The findings from this study will provide more accurate estimates of the diagnostic ability of ACL tests, to inform clinicians in these common clinical situations.

## Materials and methods

The study was registered on PROSPERO (CRD42021231446). A Preferred Reporting Items for Systematic Review and Meta-Analyses (PRISMA) protocol [51] defined the aim, objectives, the ‘Population, Intervention, Comparison, Outcomes and Study design’ (PICOS) [3] framework, search terms, inclusion and exclusion criteria.

### Search strategy

A search was conducted for relevant studies without restriction on date of publication using the bibliographic databases PubMed, Scopus, MEDLINE and Web of Science (Supplemental Table 1).

### Study selection

**Fig. 1:**
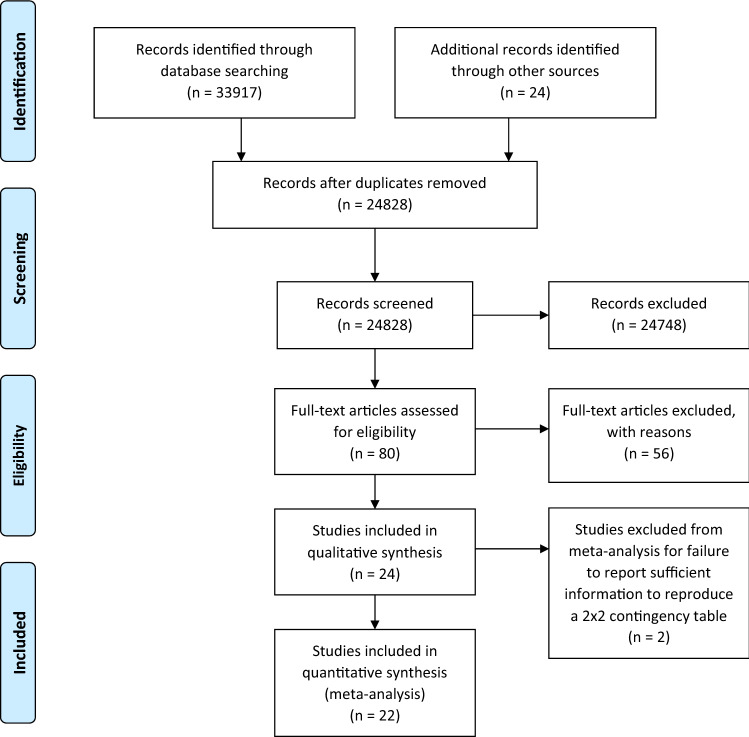
PRISMA flow diagram showing selection process outcome. Retrieved studies were systematically excluded against the inclusion and exclusion criteria. Arrows pointing downwards indicate the process of identifying relevant articles. Arrows pointing to the right show articles excluded from the review

Results from bibliographic databases were combined and duplicates removed. Studies obtained through screening previous systematic reviews were also considered and PRISMA flow diagram and checklist followed to screen literature and report selection of relevant studies (Fig. 1 and Supplemental Fig. 1) [51].

### Identification of eligible studies

Inclusion and exclusion criteria for the study are defined in Supplemental Table 2. Bibliographic database search results were uploaded into Rayyan QCRI web application (https://rayyan.qcri.org/cite) [56] and the titles and abstracts of every citation screened to exclude clearly irrelevant studies. Remaining citations were independently reviewed by PAS, RAO and RN for eligibility based on title, abstract and full text and conflicts resolved by consensus.

### Quality assessment

Standardised assessment of the quality of reporting by primary diagnostic accuracy studies and risk of bias regarding applicability of results was determined using QUADAS-2 tool [78] (Supplemental Table 3).

### Data abstraction

Values of 2 × 2 contingency tables [number of true positives (TP), false positives (FP), true negatives (TN) and false negatives (FN)] were extracted from each study. If this information was not provided, the values were calculated from descriptive statistics presented within the study. If this was not possible for at least one of the two diagnostic properties of sensitivity [TP/(TP + FN)] or specificity [TN/(TN + FP)] the studies were excluded from meta-analysis. A cut-off of 3-weeks post-injury was used to differentiate acute (< 3 weeks) and post-acute (> 3 weeks) presentations.

### Data analysis

Bivariate diagnostic random-effects meta-analysis was performed where studies reported both sensitivity and specificity as this approach is considered more valid than univariate analysis [19, 65]. A univariate random effects model meta-analysis was used where studies reported only sensitivity or specificity and to enable meaningful comparison with previous systematic reviews [65]. The sensitivity and specificity were analysed by subgroups of the time since injury (acute or post-acute), injury type (partial or complete tear) and reference used (arthroscopy or MRI). Positive (LR +) and negative (LR−) likelihood ratios were calculated from the values of sensitivity and specificity to determine the pre- to post-test shift in probability of an ACL tear [48]. The analysis was performed in R Studio (https://rstudio.com; supplemental information) [61] using mada [19] and meta [65] packages.

## Results

### Selection of studies

The total number of citations retrieved from the bibliographic databases was: PubMed 7369, Scopus 17125, MEDLINE 5094 and Web of Science 4329. Screening of previous systematic reviews identified 24 additional citations. Screening titles and abstracts of the citations against the inclusion and exclusion criteria identified 80 studies for potential inclusion with in-depth scrutiny of each article generating 24 studies for final inclusion in this review [6, 8, 9, 11, 12, 18, 20, 22, 25, 26, 33, 38, 39, 41–45, 49, 54, 57, 62, 72, 75]. Selection of studies is summarised in the PRISMA flow diagram (Fig. 1) and details of excluded studies with justifications provided in supplemental information.

### Study characteristics

Comprehensive description of each study and patient cohorts is provided in Tables 1 and 2. Only two studies [6, 8] were not considered at risk of bias (Table 3). One study was an RCT [6], seven studies had a prospective design [8, 42, 43, 49, 54, 72, 75] and 14 assessed patients retrospectively [9, 12, 18, 20, 22, 26, 33, 38, 39, 41, 44, 45, 57, 62]. Arthroscopy and/or arthrotomy was used as a gold standard in 17 studies [6, 8, 9, 11, 12, 18, 20, 22, 25, 38, 39, 43, 44, 54, 57, 62, 72] with MRI reported as a reference standard in seven studies [26, 38, 41, 42, 45, 49, 75]. Isolated ACL tears were analysed in seven studies [6, 9, 11, 20, 25, 42, 49], whereas ten studies recruited patients with an isolated ACL tear or combined ACL tear and meniscal injury [8, 18, 20, 22, 26, 39, 41, 62, 72, 75]. The spectrum of conditions was not specified or unclear in eight studies [12, 18, 33, 38, 43–45, 57]. Patient cohorts with complete ACL tears only were analysed by ten studies [6, 8, 11, 12, 20, 25, 39, 42, 44, 75], partial ACL tears only were analysed in two studies [22, 42], combined complete and partial ACL tears in nine studies [9, 18, 22, 26, 38, 43, 45, 49, 75]. Six studies did not specify the type of ACL tear [33, 41, 54, 57, 62, 72]. The number of patients in cohorts extracted from studies ranged from six [22] to 217 [22]. The average age of patients ranged from 21 [8] to 45 [6, 39] with seven studies including patients below 16 years of age [8, 9, 20, 33, 45, 49, 62]. Time from injury was not defined in three studies [38, 45, 72] while acute, subacute and/or chronic injuries were included in the same cohort in six studies [9, 33, 41, 43, 57, 75]; three studies described patients as acute yet did not specify time from injury [8, 49] or provided only the average time [62].

**Table 1:** Study characteristics extracted from each full manuscript

Study	Stated injury onset	Injury onset category	Study design	Blinding	Haemarthrosis	Random order of tests	Index test description	Gender	Age range (average)	Duration Index test to reference standard
Blanke et al. 2020 [6]	6 weeks after injury	Non-acute	RCT	Examiner blinded to clinical history and group allocation	No data	Yes	Yes	62 M 38 F	No data (ACL rupture -26.4 Controls—45.4)	No data
Bomberg et al. 1990 [8]	“acute”	Acute	Prospective	No data	Present – no mention of action	No data	No	31 M 14 F	13–42 (21)	No data
Chong et al. 2017 [9]	After 72 h	Unclear/ both	Retrospective	No data	No data	No	Yes	21 M 12 F	11–62 (30.9)	No data
Cooperman et al. 1990 [11]	> 6 weeks from surgery	Non-acute	Prospective study on retrospectively diagnosed patients	Examiner blinded to patient’s face and torso. Patients wore the type of same shorts	No data	Yes	Yes	18 M 14 F	18–57 (26)	At least 6 weeks before the study
Dahlstedt et al. 1989 [12]	< 22 days	Acute	Retrospective	No data	No data	No data	No	20 M 21 F	14–47 (27.5)	No data
Deveci et al. 2015[18]	4–25 weeks	Non-acute	Retrospective	No data	No data	No data	Lever test only	96 M 21 F	17–45 (25.8)	4–25 weeks
Donaldson et al. 1985 [20]	< 19 days	Acute	Retrospective	No data	No data	No data	Lachman only	62 M 38 F	15–49 (No data)	Within 19 days
Fok et al. 2014[22]	> 1 month	Non-acute	Retrospective	No data	No data	No data	No data	189 M 36 F	16–52 (26.4)	1–47 months
Geraets et al. 2015 [25]	> 5 months	Non-acute	Prospective study on retrospectively diagnosed patients	Blinded to clinical data	No data	Yes	Yes	41 M 19 F	19–70 (37)	No data
Gurpinar et al. 2019 [26]	< 2 weeks	Acute	Retrospective	Not blinded	Drained before examination	No	Yes	69 M 9F	17–44 (26.17)	No data
Jarbo et al. 2017 [33]	< 4 weeks	Unclear/ both	Retrospective	Examiner blinded to clinical history, scans and randomly assigned tester	No data	No	Yes	58 M 44 F	15–66 (No data)	No data
Kostov et al. 2014 [38]	Unclear	Unclear/ both	Retrospective	No data	No data	No data	Yes	81 M 22 F	16–58 (29.7)	No data
Krakowski et al. 2019 [39]	mean 44 months	Non-acute	Retrospective	Not blinded	No data	No data	Yes	47 M 49 F	No data (45)	44 months average
Lee et al. 1988 [41]	“most of our patients are nonacute”	Unclear/ both	Retrospective	No data	No data	No data	Yes	No data	14–59 (No data)	Arthroscopy within 1–5 weeks after MRI
Lelli et al. 2016 [42]	Acute – less than 20 days Chronic – 20 days to 4 years	Acute & Non-acute	Prospective	Blinded to MRI scan if available	No data	No data	Lever test only	281 M 119 F	No data (26.4)	No data
Lichtenberg et al. 2018 [43]	Acute, subacute and chronic (< 3; 3–11; > 12 weeks)	Unclear/ both	Prospective	Not blinded	No data	No	Yes	57 M 37 F	No data (34)	No data
Liu et al. 1995 [44]	Within 7 days	Acute	Retrospective	No data	No data	No data	Lachman only	27 M 11 F	16–35 (26)	Surgery within 3 weeks
Makki et al. 2019 [45]	Unclear	Unclear/ both	Retrospective	No data	If present, joint aspiration performed	N/A – 1 test	No data	44 M 26 F	13–52 (28)	No data
McQuivey et al. 2019 [49]	“acute”	Acute	Prospective	Not blinded to clinical history	No data	N/A – 1 test	Yes	14 M 7 F	12–54 (31.2)	No data
Mulligan et al. 2015 [54]	> 30 weeks	Non-acute	Prospective	Examiner blinded to clinical history, MRI scan but not to the patient’s face	No data	Yes	Yes	21 M 34 F	20–64 (40.7)	No data
Peeler et al. 2010 [57]	From 0 to 2143 days	Unclear/ both	Retrospective	No data	No data	No data	Yes	No data	18–55 (No data)	“significant time elapsed”
Sandberg et al. 1986 [62]	Average 7 days after injury	Acute	Retrospective	No data	Joint aspiration performed	No data	ADS & Lachman only	No data	13–59 (27)	No data
Thapa et al. 2015 [72]	Unclear	Unclear/ both	Prospective	No data	No data	No data	Lever test only	50 M 30 F	21–42 (32)	No data
Wagemakers et al. 2010 [75]	9–81 days	Unclear/ both	Prospective	Examiner blinded to MRI and clinical history	No data	No data	Yes	74 M 60 F	No data (40.2)	Immediate– test after MRI

The table reports data extracted from each study relevant to the study questions. *RCT* randomised controlled trial, *M* male, *F* female, *N/A* not applicable, *ADS* anterior drawer sign

**Table 2:** Patient cohorts from studies

Study	Injury onset	Complete or partial	Injury type	Reference standard	Test	Number of patients	TP	TN	FP	FN	SN	SP
Blanke et al. 2020 [6]	Non-acute	Complete	Isolated ACL	A	Lachman	100 (200)	74	83	17	26	0.74	0.83
	Non-acute	Complete	Isolated ACL	A	Pivot	100 (200)	46	96	4	54	0.46	0.96
Bomberg et al. 1990 [8]	Acute	Complete	Isolated ACL ± meniscal tear	A	ADS	21	2	0	0	19	0.10	–
	Acute	Complete	Isolated ACL ± meniscal tear	A	Lachman	21	18	0	0	3	0.86	–
	Acute	Complete	Isolated ACL ± meniscal tear	A	Pivot	21	9	0	0	12	0.43	–
Chong et al. 2017 [9]	Unclear/ Both	C & P	Isolated ACL	A	Lachman (physician assistant)	33	22	0	0	11	0.67	–
	Unclear/ Both	C & P	Isolated ACL	A	Lachman (surgeon)	33	31	0	0	2	0.94	–
	Unclear/ Both	C & P	Isolated ACL	A	Lever (physician assistant)	33	27	0	0	6	0.82	–
	Unclear/ Both	C & P	Isolated ACL	A	Lever (surgeon)	33	29	0	0	4	0.88	–
	Unclear/ Both	C & P	Isolated ACL	A	Pivot (physician assistant)	33	3	0	0	30	0.09	–
	Unclear/ Both	C & P	Isolated ACL	A	Pivot (surgeon)	33	9	0	0	24	0.27	–
Cooperman et al. 1990 [11]	Non-acute	Complete	Isolated ACL	A	Lachman (Trial 1)	32	20	19	19	6	0.77	0.50
	Non-acute	Complete	Isolated ACL	A	Lachman (Trial 2)	32	22	22	17	4	0.85	0.56
Dahlstedt et al. 1989 [12]	Acute	Complete	Unclear	A	Lachman	23	23	0	0	0	1.00	–
	Acute	Complete	Unclear	A	Pivot	23	2	0	0	21	0.09	–
Deveci et al. 2015 [18]	Unclear/ Both	C & P	Isolated ACL ± meniscal tear	A	ADS	117	70	0	0	47	0.60	–
	Unclear/ Both	C & P	Isolated ACL ± meniscal tear	A	Lachman	117	94	0	0	23	0.80	–
	Unclear/ Both	C & P	Isolated ACL ± meniscal tear	A	Lever	117	110	0	0	7	0.94	–
	Unclear/ Both	C & P	Isolated ACL ± meniscal tear	A	Pivot	117	73	0	0	44	0.62	–
Donaldson et al. 1985 [20]	Acute	Complete	Isolated ACL ± meniscal tear	A	ADS	33	25	0	0	8	0.76	–
	Acute	Complete	Isolated ACL ± meniscal tear	A	Lachman	33	33	0	0	0	1.00	–
	Acute	Complete	Isolated ACL ± meniscal tear	A	Pivot	33	14	0	0	19	0.42	–
	Acute	Complete	Isolated ACL	A	ADS	37	20	0	0	17	0.54	–
	Acute	Complete	Isolated ACL	A	Lachman	37	36	0	0	1	0.97	–
	Acute	Complete	Isolated ACL	A	Pivot	37	10	0	0	27	0.27	–
Fok et al. 2014 [22]	Non-acute	Partial	Isolated ACL ± meniscal tear	A	Pivot	6	5	0	0	1	0.83	–
	Non-acute	Partial	Isolated ACL ± meniscal tear	A	Lachman	8	6	0	0	2	0.75	–
	Non-acute	C & P	Isolated ACL ± meniscal tear	A	Pivot	206	204	0	0	2	0.99	–
	Non-acute	C & P	Isolated ACL ± meniscal tear	A	Lachman	217	205	0	0	12	0.94	–
Geraets et al. 2015 [25]	Non-acute	Complete	Isolated ACL	A	Lachman (primary care physician)	60	12	26	12	10	0.55	0.68
	Non-acute	Complete	Isolated ACL	A	Lachman (surgeon)	60	12	38	0	10	0.55	1.00
Gurpinar et al. 2019 [26]	Acute	C & P	Isolated ACL ± meniscal tear	MRI	ADS	78	48	11	5	14	0.77	0.69
	Acute	C & P	Isolated ACL ± meniscal tear	MRI	Lachman	78	50	10	6	12	0.81	0.63
	Acute	C & P	Isolated ACL ± meniscal tear	MRI	Lever	78	57	15	1	5	0.92	0.94
	Acute	C & P	Isolated ACL ± meniscal tear	MRI	Pivot	78	32	15	1	30	0.52	0.94
Jarbo et al. 2017 [33]	Unclear/ Both	Unclear	Unclear	MRI	Lever	48	10	32	1	5	0.67	0.97
Kostov et al. 2014 [38]	Unclear/ Both	C & P	Unclear	A	ADS	103	69	30	0	4	0.95	1.00
	Unclear/ Both	C & P	Unclear	A	Lachman	103	67	30	0	6	0.92	1.00
	Unclear/ Both	C & P	Unclear	A	Pivot	103	45	29	1	28	0.62	0.97
	Unclear/ Both	C & P	Unclear	MRI	ADS	103	59	28	10	6	0.91	0.74
	Unclear/ Both	C & P	Unclear	MRI	Lachman	103	58	29	9	7	0.89	0.76
	Unclear/ Both	C & P	Unclear	MRI	Pivot	103	42	34	4	23	0.65	0.89
Krakowski et al. 2019 [39]	Non-acute	Complete	Isolated ACL ± meniscal tear	A	ADS	96	–	–	–	–	0.69	0.93
	Non-acute	Complete	Isolated ACL ± meniscal tear	A	Lachman	96	27	0	0	5	0.84	0.92
	Non-acute	Complete	Isolated ACL ± meniscal tear	A	Lever	96	–	–	–	–	0.63	0.98
	Non-acute	Complete	Isolated ACL ± meniscal tear	A	Pivot	96	–	–	–	–	0.43	0.98
Lee et al. 1988 [41]	Unclear /Both	Unclear	Isolated ACL ± meniscal tear	A	ADS	41	14	23	0	4	0.78	1.00
	Unclear/ Both	Unclear	Isolated ACL ± meniscal tear	A	Lachman	41	16	23	0	2	0.89	1.00
	Unclear/ Both	Unclear	Isolated ACL ± meniscal tear	MRI	ADS	79	18	56	0	5	0.78	1.00
	Unclear/ Both	Unclear	Isolated ACL ± meniscal tear	MRI	Lachman	79	21	56	0	2	0.91	1.00
Lelli et al. 2016 [42]	Acute	Complete	Isolated ACL	MRI	ADS	100	75	0	0	25	0.75	–
	Acute	Complete	Isolated ACL	MRI	Lachman	100	66	0	0	34	0.66	–
	Acute	Complete	Isolated ACL	MRI	Lever	100	100	0	0	0	1.00	–
	Acute	Complete	Isolated ACL	MRI	Pivot	100	23	0	0	77	0.23	–
	Non-acute	Complete	Isolated ACL	MRI	ADS	100	100	0	0	0	1	–
	Non-acute	Complete	Isolated ACL	MRI	Lachman	100	100	0	0	0	1	–
	Non-acute	Complete	Isolated ACL	MRI	Lever	100	100	0	0	0	1	–
	Non-acute	Complete	Isolated ACL	MRI	Pivot	100	98	0	0	2	0.98	–
	Acute	Partial	Isolated ACL	MRI	ADS	100	29	0	0	71	0.29	–
	Acute	Partial	Isolated ACL	MRI	Lachman	100	42	0	0	58	0.42	–
	Acute	Partial	Isolated ACL	MRI	Lever	100	100	0	0	0	1.00	–
	Acute	Partial	Isolated ACL	MRI	Pivot	100	11	0	0	89	0.11	–
	Non-acute	Partial	Isolated ACL	MRI	ADS	100	83	0	0	17	0.83	–
	Non-acute	Partial	Isolated ACL	MRI	Lachman	100	39	0	0	61	0.39	–
	Non-acute	Partial	Isolated ACL	MRI	Lever	100	100	0	0	0	1	–
	Non-acute	Partial	Isolated ACL	MRI	Pivot	100	56	0	0	44	0.56	–
Lichtenberg et al. 2018 [43]	Unclear/ Both	C & P	Unclear	A	Pivot	81	–	–	–	–	0.50	0.98
	Unclear/ Both	C & P	Unclear	A	Lever	87	–	–	–	–	0.39	1
	Unclear/ Both	C & P	Unclear	A	ADS	91	–	–	–	–	0.71	0.94
	Unclear/ Both	C & P	Unclear	A	Lachman	93	–	–	–	–	0.87	0.91
Liu et al. 1995 [44]	Acute	Complete	Unclear	A	ADS	38	23	0	0	15	0.61	–
	Acute	Complete	Unclear	A	Lachman	38	36	0	0	2	0.95	–
	Acute	Complete	Unclear	A	Pivot	38	27	0	0	11	0.71	–
Makki et al. 2019 [45]	Unclear/ Both	C & P	Unclear	MRI	Lachman	50	11	28	7	4	0.73	0.80
McQuivey et al. 2019 [49]	Acute	C & P	Isolated ACL	MRI	Lever	21	3	17	1	0	1.00	0.94
Mulligan et al. 2015 [54]	Non-acute	Unclear	Unclear	A	Lachman	17	14	1	0	2	0.88	1.00
Peeler et al. 2010 [57]	Unclear/ Both	Unclear	Unclear	A	ADS (physician)	unclear	–	–	–	–	0.33	–
	Unclear/ Both	Unclear	Unclear	A	ADS (surgeon)	unclear	–	–	–	–	0.39	–
	Unclear/ Both	Unclear	Unclear	A	ADS (therapist)	unclear	–	–	–	–	0.36	–
	Unclear/ Both	Unclear	Unclear	A	Lachman (physician)	unclear	–	–	–	–	0.53	–
	Unclear/ Both	Unclear	Unclear	A	Lachman (surgeon)	unclear	–	–	–	–	0.86	–
	Unclear/ Both	Unclear	Unclear	A	Lachman (therapist)	unclear	–	–	–	–	0.27	–
	Unclear/ Both	Unclear	Unclear	A	Pivot (physician)	unclear	–	–	–	–	0.15	–
	Unclear/ Both	Unclear	Unclear	A	Pivot (surgeon)	unclear	–	–	–	–	0.63	–
	Unclear/ Both	Unclear	Unclear	A	Pivot (therapist)	unclear	–	–	–	–	0.00	–
Sandberg et al. 1986 [62]	Acute	Unclear	Isolated ACL ± meniscal tear	A	ADS	92	37	0	0	55	0.40	–
	Acute	Unclear	Isolated ACL ± meniscal tear	A	Lachman	92	40	0	0	52	0.43	–
	Acute	Unclear	Isolated ACL ± meniscal tear	A	Pivot	92	4	0	0	88	0.04	–
Thapa et al. 2015 [72]	Unclear/ Both	Unclear	Isolated ACL ± meniscal tear	A	ADS	80	28	42	3	7	0.80	0.93
	Unclear/ Both	Unclear	Isolated ACL ± meniscal tear	A	Lachman	80	32	43	2	3	0.91	0.96
	Unclear/ Both	Unclear	Isolated ACL ± meniscal tear	A	Lever	80	30	40	5	5	0.86	0.89
	Unclear/ Both	Unclear	Isolated ACL ± meniscal tear	A	Pivot	80	18	45	0	17	0.51	1.00
Wagemakers et al. 2010 [75]	Unclear/ Both	Complete	Isolated ACL ± meniscal tear	MRI	ADS	64	15	26	21	2	0.88	0.55
	Unclear/ Both	C & P	Isolated ACL ± meniscal tear	MRI	ADS	64	23	20	16	5	0.82	0.56

Characterisation of patient cohorts within studies

*C & P* complete and partial, *ADS* anterior drawer sign, *A* arthroscopy, *MRI* Magnetic Resonance Imaging, *ACL* anterior cruciate ligament, *SN* sensitivity, *SP* specificity, *TP* true positives, *TN* true negatives, *FP* false positives, *FN* false negatives

**Table 3:**
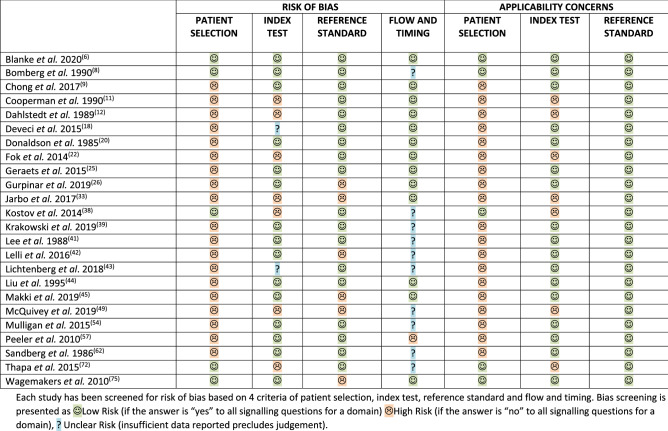
QUADAS-2 tool assessment shows potential risks based on study’s design

Each study has been screened for risk of bias based on 4 criteria of patient selection, index test, reference standard and flow and timing. Bias screening is presented as Low Risk (if the answer is “yes” to all signalling questions for a domain) High Risk (if the answer is “no” to all signalling questions for a domain), ? Unclear Risk (insufficient data reported precludes judgement)

Two studies performed arthrocentesis before examination [26, 62] and two studies did not aspirate the joint, while the remaining studies make no mention of such interventions. Six studies implemented measures to blind the examiner to patient history [6, 11, 25, 33, 54, 75] and available imaging [6, 11, 25, 33, 42, 54, 75] or to prevent facial recognition of a previously seen patient [11], whilst other studies did not report and/or did not use such strategies. Random order of testing was applied in four studies [6, 11, 25, 54].

### Meta-analysis

**Fig. 2:**
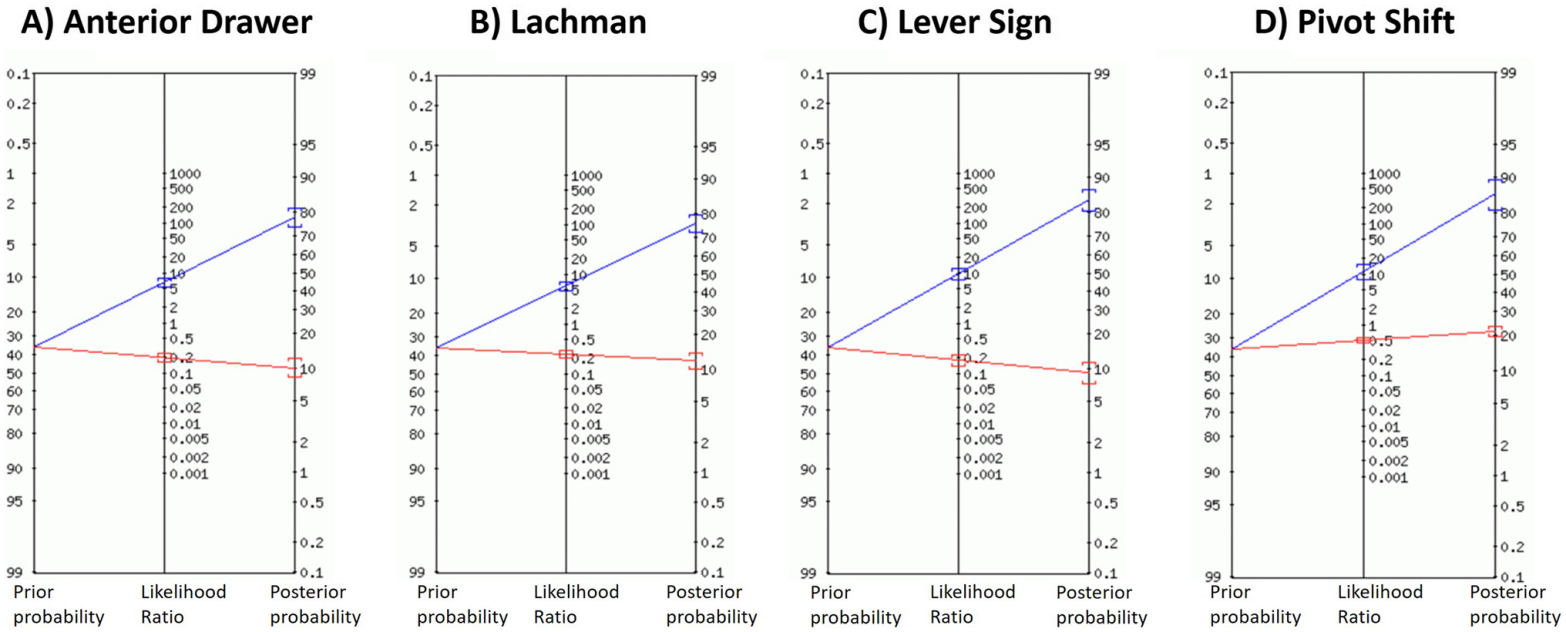
Fagan’s nomogram of shift in pre-test to post-test probability of clinical tests. Fagan’s nomograms illustrating the shift in pre-test to post-test probability for the **a** anterior drawer, **b** Lachman, **c** Lever sign and **d** Pivot shift tests. The pre-test probability of acute ACL tear is shown on the left vertical axis (36%). The post-test probability is shown on the right vertical axis. The middle vertical axis shows value of likelihood ratio. The blue line represents a change in ACL tear probability when the test is positive. The red line indicates a change in ACL injury probability when test is negative

Bivariate random effects model meta-analysis was possible for data from 12 studies (Table 4 and Supplemental Tables 4, 5, 6) [6, 11, 25, 26, 33, 38, 41, 45, 49, 54, 72, 75]. For all studies combined, the pivot shift test had the highest specificity (94%) and LR + (10.70), producing a large shift in the positive post-test probability. However, this test also had the lowest sensitivity (55%) and highest LR− (0.48), producing the smallest shift in negative post-test probability. The Lever sign produced moderate shifts in the positive post-test probability and was the only test to produce a moderate shift in the negative post-test probability. The anterior drawer and Lachman tests produced moderate shifts in the positive post-test probability and small shifts in the negative post-test probability. The post-test probability of an ACL tear for all tests is illustrated in Fig. 2 using a 36% pre-test probability [63].

**Table 4:** Univariate and bivariate analysis of diagnostic clinical tests for all studies evaluated

	All studies [95% CI]				
	Sn	Sp	LR+	LR−	AUC
Anterior Drawer (UA)	0.75 [0.61; 0.86]	0.92 [0.67; 0.99]	2.4 [1.58; 3.64]	0.28 [0.20; 0.42]	–
Anterior Drawer (BA)	0.83 [0.77; 0.88]	0.85 [0.64; 0.95]	6.34 [2.32; 15.30]	0.20 [0.14; 0.30]	0.87
Lachman (UA)	0.85 [0.77; 0.91]	0.93 [0.77; 0.98]	2.72 [1.97; 3.77]	0.27 [0.20; 0.36]	–
Lachman (BA)	0.81 [0.73; 0.87]	0.85 [0.73; 0.92]	5.72 [2.82; 10.80]	0.24 [0.15; 0.35]	0.882
Lever Sign (UA)	0.98 [0.88; 1.00]	0.93 [086; 0.96]	4.56 [2.79; 7.45]	0.15 [0.09; 0.26]	–
Lever Sign (BA)	0.83 [0.68; 0.92]	0.91 [0.83; 0.95]	9.66 [5.01;17.30]	0.18 [0.09; 0.34]	0.938
Pivot Shift (UA)	0.48 [0.29; 0.68]	0.96 [0.92; 0.98]	1.45 [0.73; 2.87]	0.52 [0.43; 0.64]	–
Pivot Shift (BA)	0.55 [0.47; 0.62]	0.94 [0.88; 0.97]	10.70 [5.43; 19.30]	0.48 [0.40; 0.56]	0.828

*AUC* area under the curve, *BA* bivariate analysis, *CI* confidence interval, *LR−* negative likelihood ratio, *LR+* positive likelihood ratio, *Sn* sensitivity, *Sp* specificity, *UA* univariate analysis

Comparison of diagnostic clinical tests (anterior drawer, Lachman, Lever sign and pivot shift) in complete and partial ACL tears, acute and post-acute clinical presentations with arthroscopy and MRI as the reference standard was performed using univariate and bivariate modelling

Based on studies using gold-standard arthroscopy and/or arthrotomy for reference [6, 8, 9, 11, 12, 18, 20, 22, 25, 38, 39, 43, 44, 54, 57, 62, 72], the anterior drawer was the most accurate test with the highest LR+ (25.10) and joint lowest LR− (0.17) alongside the Lever sign, but LR+ confidence intervals were wide. The pivot shift test produced large shifts in the positive post-test probability, with the Lachman and Lever sign producing moderate shifts. The anterior drawer and Lever sign tests produced moderate shifts in the negative post-test probability, with the Lachman and pivot shift tests producing small shifts.

Where MRI was used as the reference standard (Supplemental Table 4) [26, 38, 41, 42, 45, 49, 75], the Lever sign was the most accurate test with the highest LR+ (13.50) and lowest LR− (0.20). The specificity and LR + of the anterior drawer and LR + of the pivot shift were considerably lower than values reported using arthroscopy and/or arthrotomy.

For specific presentations, bivariate random effects model meta-analysis was only possible for the Lachman test in post-acute injuries and complete ACL tears. In both presentations, the Lachman test produced small shifts in the positive and negative post-test probability (Supplemental Tables 5 and 6).

Pooled diagnostic accuracy estimates were determined with univariate analyses of data from 23 studies [6, 8, 9, 11, 12, 18, 20, 22, 25, 26, 33, 38, 39, 41–45, 49, 54, 57, 72, 75] (Table 4 and Supplemental Tables 4, 5, 6). In acute presentations (< 3 weeks since injury), the Lever sign was the most accurate test with the highest sensitivity (100%) and joint highest specificity (94%) alongside the pivot shift. In post-acute presentations (> 3 weeks since injury), the Lever sign had the highest sensitivity (100%), with the anterior drawer and pivot shift tests demonstrating considerably higher sensitivity values than in acute presentations. The Lachman test’s specificity was also higher than in acute presentations; insufficient data were available to calculate specificity values for the anterior drawer or Lever sign tests.

For studies reporting complete ACL tears only (Supplemental Table 5), the pivot shift test demonstrated the highest specificity (96%) but lowest sensitivity (48%). The Lever sign and Lachman tests had the highest sensitivity, with Lachman also demonstrating high specificity; insufficient data were available to determine the Lever sign test’s specificity in complete tears. For partial ACL tears, sensitivity values only were available for all four tests (Supplemental Table 5). With exception of the Lever sign, all tests demonstrated inferior diagnostic ability for ruling out partial ACL tears compared with complete ruptures.

## Discussion

The most important finding of the present study was the lower diagnostic accuracy values for the Lachman test compared with those reported in previous systematic reviews [5, 40, 74]. Based on a bivariate analysis of all studies combined, the pivot shift and Lever sign were the best tests overall for ruling in or ruling out an ACL tear, respectively. The diagnostic accuracy values between the anterior drawer, Lachman and Lever sign tests were comparable, but the Lachman test demonstrated lower accuracy in post-acute presentations and complete ACL tears alone. However, results should be interpreted with caution due to limited quality evidence and heterogeneity of included studies.

To date, ten previous systematic reviews have been conducted to determine the diagnostic accuracy of clinical tests for ACL injury [2, 5, 29, 31, 40, 59, 64, 67, 70, 74]. Eight reviews compared the anterior drawer, Lachman, and pivot shift tests [5, 29, 31, 40, 64, 67, 70, 74], with the remaining two reviews reporting on the Lever sign test alone [2, 59]. The highest quality meta-analyses for pertinent tests utilised univariate approaches [2, 5, 59], while the only meta-analysis employing a bivariate method [64] reported diagnostic estimates for the anterior drawer and Lachman tests alone without corresponding likelihood ratios [64], limiting comparison between studies. The current systematic review with meta-analysis is therefore the first to provide pooled diagnostic estimates using bivariate random effects models for the pivot shift and Lever sign tests overall, and the Lachman test in specific presentations. This study is also the first to compare all four tests directly using the same criteria, report the diagnostic accuracy of the Lever sign test in acute and post-acute presentations, and includes several new studies.

With exception of Leblanc et al. [40], which excluded studies published before the year 2000, previous systematic reviews included subjects with concomitant knee ligament injury [4, 7, 28, 34, 47, 50, 77]. In an ACL deficient knee, concomitant medial or lateral knee injuries can decrease or increase the grading of the pivot shift test respectively [69, 71] or preclude the pivot shift phenomenon from taking place altogether [32]. Likewise, the anterior drawer and Lachman tests are thought to become increasingly positive if secondary restraints to anterior tibial translation are also injured [5, 30, 35]. No studies have validated the pathomechanics of the Lever sign [33], therefore it is unknown whether additional ligament injury influences the outcome of this test. Since concomitant ligament injuries may affect the sensitivity of an ACL test, the current study provides valuable information regarding the diagnostic accuracy of ACL tests in the absence of such injuries.

### Comparison with other studies

Benjaminse et al. [5], the highest quality meta-analysis with the most studies included [14], employed a univariate approach and describe Lachman as the most valid test overall (Sn:85%, Sp:94%). An equivalent univariate analysis performed in the current study demonstrates almost identical values (Sn:85%, Sp:93%) (Table 4) but bivariate analysis, which provides a more accurate estimate of pooled effect sizes, indicates lower diagnostic accuracy. Since Benjaminse et al. [5] included studies with concomitant knee ligament injury and the current review excluded such studies, concomitant ligament injury may not necessarily affect the validity of the Lachman test, but its diagnostic accuracy may have been previously overestimated. Future studies should directly compare the diagnostic accuracy of tests in ACL-injured patients with and without additional ligament injury.

The findings of the present study support recommendations that the pivot shift should be used to rule in an ACL tear when positive [5, 14] but the Lachman test did not demonstrate superior validity when compared with the anterior drawer or Lever sign; therefore, the hypotheses were rejected. Specifically, the diagnostic accuracy of the Lachman test is considerably lower than previously reported in post-acute presentations [5] and complete tears [40] based on bivariate analysis values.

### Lever sign caution

The lever sign appears to be an accurate test regardless of time since injury or tear type, somewhat refuting the interdependence between sensitivity and specificity. However, it is worth noting that the only study with moderate methodology reported a sensitivity of 68% [33] and most studies demonstrate limited quality [2, 59]. Reiman et al. [59] reported two diagnostic accuracy values, one including and the other excluding data from the original study [42]. With the original data omitted (400 tests with no false positive or false negative results), the LR + decreased from 128.0 to 13.1 indicating verification and case–control bias [59]. Other studies reporting on the Lever sign test are also at risk of verification bias [9, 18, 26, 33, 39, 43, 49, 72]; therefore, results based on time since injury and tear type should be interpreted with caution.

The original Lever sign study data [42] were included in univariate analyses but excluded from bivariate analyses; the latter demonstrates inferior diagnostic values for the Lever sign test, yet it is still comparable with the other tests. Although bivariate analysis of MRI studies identifies the Lever sign as the most accurate test for diagnosing ACL tears, the diagnostic accuracy of MRI is dependent on magnetic field strength [58, 66] and arthroscopic assessment remains the gold standard; therefore, results should be interpreted accordingly. Given that the Lever sign is the only test to produce a moderate shift in the negative post-test probability, other modalities (i.e., arthrometry, MRI or arthroscopy) should be considered when the history is suggestive of an ACL injury, but clinical tests are negative.

### Strengths, limitations and recommendations

To ensure this review was as clinically relevant as possible, only data for clinical tests performed on non-anaesthetised (awake) patients, without additional measuring equipment (e.g., arthrometry), were included. PRISMA guidelines and QUADAS-2 risk of bias analysis were used to promote methodological quality of the study. Where possible, a bivariate random effects model meta-analysis was performed as this method provides the most accurate estimate of pooled effect sizes and is recommended for meta-analysis of diagnostic test accuracy [27]. However, only 12 studies qualified for bivariate analysis and a lack of sufficient data precluded comparison between tests based on time since injury and tear type. Further research is required to establish more accurate estimates for the Lachman test in acute presentations and partial ACL tears using bivariate analysis.

Univariate analyses were performed where bivariate analysis was not possible and to allow comparison with previous systematic reviews [2, 5, 59], but the difference between methods should be acknowledged. For example, univariate analyses in post-acute presentations and complete ACL tears demonstrate higher sensitivity and specificity values for the Lachman test than bivariate methods, thereby overestimating the test’s diagnostic accuracy. The likelihood ratios calculated by univariate analysis also cast doubt on the accuracy and reliability of these values; therefore, results should be interpreted with caution.

The methodological quality of many studies was compromised by numerous factors including a retrospective design and lack of examiner blinding from clinical information that could bias the test outcome. With specific reference to the Lever sign test, studies should clearly report the landmarks chosen for hand placement, surface used (hard or soft), fist size, calf size and softness, all of which could affect test outcomes [33, 39, 42, 47]. Future studies should comply with the STARD guidelines [10] for completeness and transparency of reporting.

For this review, arbitrary time frames were used to differentiate acute and post-acute injuries. Whilst the terminology and time frames are a subject of debate, these categories were applied based on the most frequently reported thresholds amongst included studies. In addition to time since injury, future studies should report other covariables that could influence the outcome of a test. For example, a patient that is examined 4 weeks after injury may no longer be categorised as ‘acute’ but can still present with impairments that impact the examiner’s ability to perform a test unequivocally (e.g., pain, effusion, protective guarding). It is proposed that presentations should be differentiated not only by the time since injury, but also the presence or absence of associated impairments. This has previously been suggested to improve patient care following injury [36].

Studies with concomitant ligament injury were excluded from this systematic review but those reporting non-obstructive meniscal tears were included. Meta-regression analysis demonstrated increased sensitivity of the Lachman test with a concomitant meniscal tear, but no difference for the pivot shift or Lever sign tests. No studies reported meniscal root tears or ramp lesions, which have been shown to increase anterior and rotational laxity in an ACL deficient knee [16, 17, 23, 53, 68]. However, the awareness and understanding of these associated lesions has improved over the last decade and they may, therefore, have been overlooked in older studies. Future studies should compare ACL test findings with and without concomitant meniscal injury, to determine their impact on diagnostic accuracy.

## Conclusion

In the absence of concomitant knee ligament injury, the pivot shift and Lever sign tests demonstrate the highest diagnostic accuracy for ruling in or ruling out an ACL tear, respectively. The anterior drawer, Lachman and Lever sign tests demonstrate similar diagnostic accuracy, but diagnostic accuracy values for the Lachman test are lower than previously reported. Within the clinical setting, other modalities (including MRI and arthrometry) are recommended when the history is suggestive of an ACL tear, but tests are negative. Where tests are positive, clinicians can diagnose an ACL tear confidently without additional assessment techniques.

## Supplementary Information

Below is the link to the electronic supplementary material.

Supplementary file1 (DOCX 26 KB)

Supplementary file2 (DOCX 22 KB)

Supplementary file3 (DOCX 13 KB)

Supplementary file4 (DOCX 14 KB)

Supplementary file5 (DOCX 14 KB)

Supplementary file6 (DOCX 14 KB)

Supplementary file7 (DOCX 14 KB)

Supplementary file8 (DOCX 14 KB)
