# Supplementary material for: The diagnostic accuracy of clinical tests for anterior cruciate ligament tears are comparable but the Lachman test has been previously overestimated: a systematic review and meta-analysis
Source: Knee Surg Sports Traumatol Arthrosc. 2022 Feb 12;30(10):3287–303. doi: 10.1007/s00167-022-06898-4 (PMC9464183; doi:10.1007/s00167-022-06898-4)
Supplement: Supplementary file 1 — Supplementary file1 (DOCX 26 KB) [file 167_2022_6898_MOESM1_ESM.docx]

**SUPPLEMENTAL INFORMATION**

**R CODES USED IN META-ANALYSIS**

- **codes for univariate Sens**

library(meta)

if the units commands are not working then use:

R>library(grid)

or

R> library(readr)

Overall

R> sensitivity_logit <- metaprop(Leveru$TP, Leveru$TP+ Leveru$FN, comb.fixed=FALSE, comb.random=TRUE, sm="PLOGIT", method.ci="CP", studlab=Leveru$Study)

R>print(sensitivity_logit, digits=2)

R>forest(sensitivity_logit, digits=2, rightcols=c("effect", "ci"), xlab="Sensitivity", sortvar = TE, plotwidth=unit(9, "cm"), colgap=unit(5, "mm"), xlim = c(0,1))

By standard (when you change the byvar value it can be by anything)

R>sensitivity_logit <- metaprop(Leveru$TP, Leveru$TP+ Leveru$FN, comb.fixed=FALSE, comb.random=TRUE, sm="PLOGIT", method.ci="CP", studlab=Leveru$Study, byvar = Leveru$Standard)

R>print(sensitivity_logit, digits=2)

R>forest(sensitivity_logit, digits=2, rightcols=c("effect", "ci"), xlab="Sensitivity", sortvar = TE, plotwidth=unit(9, "cm"), colgap=unit(5, "mm"), xlim = c(0,1))

grid.text("Anterior Drawer Sign Pooled Sensitivity", .5, .75, gp=gpar(cex=1.5))

- **codes for univariate Spec**

library(meta)

Overall

R> specificity_logit <- metaprop(Leveru$TN, Leveru$TN+ Leveru$FP, comb.fixed=FALSE, comb.random=TRUE, sm="PLOGIT", method.ci="CP", studlab=Leveru$Study)

R>print(specificity_logit, digits=2)

R>forest(specificity_logit, digits=2, rightcols=c("effect", "ci"), xlab="Specificity", sortvar = TE, plotwidth=unit(9, "cm"), colgap=unit(5, "mm"), xlim = c(0,1))

By standard (when you change the bycar value it can be by anything)

R> specificity_logit <- metaprop(Leveru$TN, Leveru$TN+ Leveru$FP, comb.fixed=FALSE, comb.random=TRUE, sm="PLOGIT", method.ci="CP", studlab=Leveru$Study, byvar = Leveru$Standard)

R>print(specificity_logit, digits=2)

R>forest(specificity_logit, digits=2, rightcols=c("effect", "ci"), xlab="Specificity", sortvar = TE, plotwidth=unit(9, "cm"), colgap=unit(5, "mm"), xlim = c(0,1))

grid.text("Anterior Drawer Sign Pooled Specificity", .5, .75, gp=gpar(cex=1.5))

- **codes for bivariate**

R> library("mada")

SROC overall

R>fit.ADS<-reitsma(ADS_BA)

R>summary(fit.ADS)

R>plot(fit.ADS, xlim=c(0,1), ylim = c(0,1),

main = "SROC curve (bivariate model) for for ADS")

R>points(fpr(ADS_BA), sens(ADS_BA), pch=2)

R>legend("bottomright", c("data", "summary estimate"), pch= c(2,1))

R>legend("bottomleft", c("SROC", "conf. region"), lwd = c(2,1))

R>text(x=0.8, y=0.6, labels="Summary estimates:

AUC = 0.87

Sensitivity = 0.834

Specificity = 0.848)

SROC comparison

R>LachA<-subset(Lachman_BA, Lachman_BA$Standard=="A")

R>LachMRI<-subset(Lachman_BA, Lachman_BA$Standard=="MRI")

R>fit.LachA<-reitsma(LachA)

R>fit.LachMRI<-reitsma(LachMRI)

R>summary(fit.LachA)

R>summary(fit.LachMRI)

R>plot(fit.LachA, xlim=c(0,1), ylim = c(0,1),

main = "Comparison of Arthroscopy and MRI as reference standard for Lachman")

R>lines(sroc(fit.LachMRI), lty=2)

R>ROCellipse(fit.LachMRI, lty=2, pch=2, add=TRUE)

R>points(fpr(LachA), sens(LachA), cex=1)

R>points(fpr(LachMRI), sens(LachMRI),pch=2, cex=1)

R>legend("bottomright", c("A", "MRI"), pch=1:2, lty = 1:2)

R>text(x=0.8, y=0.6, labels="Arthroscopy summary estimates:

AUC = 0.884

Sensitivity = 0.787

Specificity = 0.877

MRI summary estimates:

AUC = 0.875

Sensitivity = 0.844

Specificity = 0.790")

- **codes for bivariate SROC comparison**

plot(fit.ADS, xlim=c(0,1), ylim = c(0,1),main = "SROC curve (bivariate model) comparison for each test") lines(sroc(fit.Lach), lty=2, col="blue") ROCellipse(fit.Lach, lty=2, pch=2, col="blue", add=TRUE) lines(sroc(fit.Lev), lty=4, col="red") ROCellipse(fit.Lev, lty=4, pch=3, col="red", add=TRUE) lines(sroc(fit.Piv), lty=5, col="green") ROCellipse(fit.Piv, lty=5, pch=4, col="green", add=TRUE) legend("bottomright", c("ADS", "Lachman", "Lever", "Pivot"), pch=1:2:3:4, lty = 1:2:3:4)

text(x=0.8, y=0.85, labels="ADS summary estimates: AUC = 0.87 Sensitivity = 0.834 Specificity = 0.848")

text(x=0.8, y=0.65, col="blue", labels="Lachman summary estimates: AUC = 0.882 Sensitivity = 0.805 Specificity = 0.850")

text(x=0.8, y=0.45, col="red", labels="Lever summary estimates: AUC = 0.938 Sensitivity = 0.834 Specificity = 0.910")

text(x=0.8, y=0.25, col="green", labels="Pivot summary estimates: AUC = 0.828 Sensitivity = 0.548 Specificity = 0.936")

- **codes for meta-regression**

R> fit.Lach.Duration.Regression <- reitsma(Lachman_BA, formula = cbind(tsens, tfpr) ~ Duration)

R> summary(fit.Lach.Duration.Regression)

Error in qr.solve(invtUX, invtUy) : singular matrix 'a' in solve

- **another univariate LR**

library(mada)

ADSuP<-subset(ADSu, ADSu$Tear=="Partial") posLR.DSL <- madauni(ADSuP, type = "posLR", method = "DSL") summary(posLR.DSL)

**SELECTION OF STUDIES**

Evaluation of full texts resulted in 56 studies being excluded. Nineteen studies combined test results and did not provide individual test data [1,11,13,16,17,23,27-29,35,39,40-42,47,50,51,53,57], six articles did not specify what tests were used [2,7,18,36-38] and two studies were excluded for reporting equivocal test results [43,44]. Examination under anaesthesia was performed in four articles [12,15,19,26], while one study performed physical tests with the use of an assistive device [3]. Ten studies included subjects with multi-ligament injury [4,6,9,20,24,25,30,31,32,45] and one study contained only two subjects with an ACL injury [48]. One study [33] and a chronic injury cohort from another study were excluded [8] for having subjects with previous history of ACL reconstruction. Two studies were excluded for failing to provide sufficient information to generate the data required for meta-analysis [22,34], seven full-text articles could not be accessed despite attempts to contact the authors [5,14,21,46,49,54,56] and the remaining three articles were written in German [10,52,55].

1. Adalberth T, Roos H, Laurén M, Åkeson P, Sloth M, Jonsson K, Lindstrand A, Lohmander LS. (1997) Magnetic resonance imaging, scintigraphy, and arthroscopic evaluation of traumatic hemarthrosis of the knee. Am J Sports Med. 25:231-237.
2. Akdemir M, Ünal M, Tatari H, Pinar H, Karaoglan O. (2020) Comparison of physical examination and MRI for the diagnosis of intraarticular knee pathologies: analysis of 968 knees. Med dello Sport. 73:302-311.
3. Andersen HN, Frandsen PA. (1993) Assessment of anterior cruciate laxity using the Genucom System. Int Orthop. 17:375-83.
4. Anderson AF, Lipscomb AB. (1989) Preoperative instrumented testing of anterior and posterior knee laxity. Am J Sports Med. 17:387-92.
5. Babalola OR, Itakpe SE, Afolayan TH, Olusola-Bello MA, Egbekun EI. (2021) Predictive Value of Clinical and Magnetic Resonance Image Findings in the Diagnosis of Meniscal and Anterior Cruciate Ligament Injuries. West Afr J Med. 38:853-856.
6. Boeree NR, Ackroyd CE. (1991) Assessment of the menisci and cruciate ligaments: an audit of clinical practice. Injury. 22:291-294.
7. Curtin W, O’Farrell D, McGoldrick F, Dolan M, Mullan G, Walsh M. (1992) The correlation between clinical diagnosis of knee pathology and findings at arthroscopy. Ir J Med Sci. 40:104-107.
8. Dahlstedt LJ, Dalén N. (1989) Knee laxity in cruciate ligament injury: Value of examination under anesthesia. Acta Orthop. 60:181–184.
9. Decary S, Fallaha M, Belzile S, Martel-Pelletier J, Pelletier JP, Feldman D, et al. (2018) Clinical diagnosis of partial or complete anterior cruciate ligament tears using patients’history elements and physical examination tests. PLoS One. 13: e0198797.
10. Decker R, Ruf W. (1988) The diagnostic certainty of various methods of evaluating recent trauma to knee ligaments. Unfallchirurgie. 14:204-10.
11. Dejour D, Ntagiopoulos PG, Saggin PR, Panisset JC. (2013) The diagnostic value of clinical tests, magnetic resonance imaging, and instrumented laxity in the differentiation of complete versus partial anterior cruciate ligament tears. Arthrosc - J Arthrosc Relat Surg. 20:491-499.
12. Di Iorio A, Carnesecchi O, Philippot R, Farizon F. (2014) Multiscale analysis of anterior cruciate ruptures: Prospective study of 49 cases. Orthop Traumatol Surg Res. 100:751-4.
13. Dutka J, Skowronek M, Skowronek P, Dutka Ł. (2012) Arthroscopic verification of objectivity of the orthopaedic examination and magnetic resonance imaging in intra-articular knee injury. Retrospective study. Wideochirurgia I Inne Tech Maloinwazyjne. 7:13-8.
14. Elfadli M, Boshalla H, Makhlouf S, Khalil M, Noh H. (2009) Sensitivity and specificity of clinical examination, magnetic resonance imaging and arthroscopy in the diagnosis of chronic knee joint injury. Jamahiriya Med J. 9:294-296.
15. Fahmy FS, Fathi H. (2019) Lever sign test: Is it sensitive for the diagnosis of anterior cruciate ligament disruption? Curr Orthop Pract. 30:343-346.
16. Felli L, Garlaschi G, Muda A, Tagliafico A, Formica M, Zanirato A, et al. (2016) Comparison of clinical, MRI and arthroscopic assessments of chronic ACL injuries, meniscal tears and cartilage defects. Musculoskelet Surg. 100:231-238.
17. Frobell RB, Lohmander LS, Roos HP. (2007) Acute rotational trauma to the knee: Poor agreement between clinical assessment and magnetic resonance imaging findings. Scand J Med Sci Sport. 17:109-114.
18. Gelb HJ, Glasgow SG, Sapega AA, Torg JS. (1996) Magnetic resonance imaging of knee disorders: Clinical value and cost-effectiveness in a sports medicine practice. Am J Sports Med. 12:127-128.
19. Guenther D, Zhang C, Ferlin F, Vernacchia C, Musahl V, Irrgang JJ, et al. (2020) Clinical examination of partial ruptures of the anterior cruciate ligament: A retrospective case–control study. Knee. 27:1866-1873.
20. Hardaker WT, Garrett WE, Bassett FH. (1990) Evaluation of acute traumatic hemarthrosis of the knee joint. South Med J. 83:640-644.
21. Harilainen A. (1987) Evaluation of knee instability in acute ligamentous injuries. Ann Chir Gynaecol. 76:269-73.
22. Hughston JC, Andrews JR, Cross MJ, et al. (1976) Classification of knee ligament instabilities. Part II. The lateral compartment. J Bone Joint Surg Am. 58(2):173-179.
23. Jah AAE, Keyhani S, Zarei R, Moghaddam AK. (2005) Accuracy of MRI in comparison with clinical and arthroscopic findings in ligamentous and meniscal injuries of the knee. Acta Orthop Belg. 71:189-96.
24. Jain DK, Amaravati R, Sharma G. (2009) Evaluation of the clinical signs of anterior cruciate ligament and meniscal injuries. Indian J Orthop. 43:375-378.
25. Jonsson T, Althoff bo, Peterson L, Renström P. (1982) Clinical diagnosis of ruptures of the anterior cruciate ligament: A comparative study of the Lachman test and the anterior drawer sign. Am J Sports Med. 10:100-102.
26. Katz JW, Fingeroth RJ. (1986) The diagnostic accuracy of ruptures of the anterior cruciate ligament comparing the Lachman test, the anterior drawer sign, and the pivot shift test in acute and chronic knee injuries. Am J Sports Med. 4(1):88-91.
27. Khan HA, Ahad H, Sharma P, Bajaj P, Hassan N, Kamal Y. (2015) Correlation between magnetic resonance imaging and arthroscopic findings in the knee joint. Trauma Mon. 20:e18635
28. Kocabey Y, Tetik O, Isbell WM, Atay A, Johnson DL. (2004) The value of clinical examination versus magnetic resonance imaging in the diagnosis of meniscal tears and anterior cruciate ligament rupture. Arthroscopy. 20:696-70.
29. Madhusudhan TR, Kumar TM, Bastawrous SS, Sinha A. (2008) Clinical examination, MRI and arthroscopy in meniscal and ligamentous knee injuries - A prospective study. J Orthop Surg Res. 19:3:19.
30. Massey PA, Harris JD, Winston LA, Lintner DM, Delgado DA, McCulloch PC. (2017) Critical Analysis of the Lever Test for Diagnosis of Anterior Cruciate Ligament Insufficiency. Arthrosc - J Arthrosc Relat Surg. 33:1560-1566.
31. Mitsou A, Vallianatos P. (1988) Clinical diagnosis of ruptures of the anterior cruciate ligament: a comparison between the Lachman test and the anterior drawer sign. Injury. 1988; 19:427-428.
32. Makhmalbaf H, Moradi A, Ganji S, Omidi-Kashani F. (2013) Accuracy of Lachman and anterior drawer tests for anterior cruciate ligament injuries. Arch Bone Jt Surg. 1:94-97.
33. Mulligan EP, Anderson A, Watson S, Dimeff RJ. (2017) The diagnostic accuracy of the Lever sign for detecting anterior cruciate ligament injury. Int J Sports Phys Ther. 12:1057-1067.
34. Mulligan EP, Harwell JL, Robertson WJ. (2011) Reliability and diagnostic accuracy of the Lachman test performed in a prone position. J Orthop Sports Phys Ther. 2011;41(10):749-757.
35. Navali AM, Bazavar M, Mohseni MA, Safari B, Tabrizi A. (2013) Arthroscopic evaluation of the accuracy of clinical examination versus MRI in diagnosing meniscus tears and cruciate ligament ruptures. Arch Iran Med. 16:229-232.
36. Nickinson R, Darrah C, Donell S. (2010) Accuracy of clinical diagnosis in patients undergoing knee arthroscopy. Int Orthop. 34:39-44.
37. Oberlander MA, Shalvoy RM, Hughston JC. (1993) The accuracy of the clinical knee examination documented by arthroscopy. A prospective study. Am J Sports Med. 21:773-778.
38. O’Shea KJ, Murphy KP, Heekin RD, Herzwurm PJ. (1996) The diagnostic accuracy of history, physical examination, and radiographs in the evaluation of traumatic knee disorders. Am J Sports Med. 24:164-7.
39. Panigrahi R, Priyadarshi A, Palo N, Marandi H, Kumar Agrawalla D, Ranjan Biswal M. (2017) Correlation of Clinical Examination, MRI and Arthroscopy Findings in Menisco-Cruciate Injuries of the Knee: A Prospective Diagnostic Study. Arch Trauma Res. 6:e30364.
40. Patel I, Chandru V, Nekkanti S, Renukarya R, Reddy VV, Gopalakrishna SV. (2018) Clinical, Magnetic Resonance Imaging, and Arthroscopic Correlation in Anterior Cruciate Ligament and Meniscal Injuries of the Knee. J Orthop Trauma Rehabil. 24:52-56.
41. Rayan F, Bhonsle S, Shukla DD. (2009) Clinical, MRI, and arthroscopic correlation in meniscal and anterior cruciate ligament injuries. Int Orthop. 33:129-132.
42. Rose NE, Gold SM. (1996) A comparison of accuracy between clinical examination and magnetic resonance imaging in the diagnosis of meniscal and anterior cruciate ligament tears. Arthroscopy. 12(4):398-405.
43. Strand T, Solheim E. (1995) Clinical tests versus KT-1000 instrumented laxity test in acute anterior cruciate ligament tears. Int J Sports Med. 6:51-3.

1. Torg JS, Conrad W, Kalen V. (1976) Clinical I diagnosis of anterior cruciate ligament instability in the athlete. Am J Sports Med. 4:84-93.
2. Warren RF, Marshall JL. (1978) Injuries of the anterior cruciate and medial collateral ligaments of the knee. A retrospective analysis of clinical records--part I. Clin Orthop Relat Res. 136:191-197.
3. Scoz RD, Amorim CF, Mazziotti BOA, da Silva RA, Vieira ER, Lopes AD, et al. (2020) Diagnostic validity of an isokinetic testing to identify partial anterior cruciate ligament injuries. J Sport Rehabil. 29:1086-1093.
4. Shahani MA, Sah RK, Khan RA, Awais SM. (2015) Arthroscopic determination of accuracy of clinical examination in injuries with internal derangement of knee. Ann King Edward Med Univer. Lahore Pakistan 21:168-168.
5. Sharma UK, Shrestha BK, Rijal S, Bijukachhe B, Barakoti R, Banskota B, et al. (2011) Clinical, MRI and arthroscopic correlation in internal derangement of knee. Kathmandu Univ Med J. 9:174-178.
6. Siddiqui MA zfa., Ahmad I, Sabir AB i., Ullah E, Rizvi SA mja. A, Rizvi SW (2013) Clinical examination vs. MRI: evaluation of diagnostic accuracy in detecting ACL and meniscal injuries in comparison to arthroscopy. Polish Orthop Traumatol. 78:59-63.
7. Simonsen O, Jensen J, Mouritsen P, Lauritzen J. (1884) The accuracy of clinical examination of injury of the knee joint. Injury. 16:96-101.
8. Stanitski CL. (1998) Correlation of arthroscopic and clinical examinations with magnetic resonance imaging findings of injured knees in children and adolescents. Am J Sports Med. 26:2-6.
9. Steinbrück K, Wiehmann JC. (1988) Examination of the knee joint. The value of clinical findings in arthroscopic control. Z Orthop Ihre Grenzgeb. 126:289-95.
10. Syal A, Chudasama CH. (2015) Clinical examination, magnetic resonance imaging and arthroscopic correlations of ligament and menisci injuries of knee joint. J Arthrosc Jt Surg. 2:3-8.
11. Tonino AJ, Huy J, Schaafsma J. (1986) The diagnostic accuracy of knee testing in the acutely injured knee. Initial examination versus examination under anaesthesia with arthroscopy. Acta Orthop Belg. 52:479-487.
12. Wirth CJ, Kolb M. (1985) Hemarthrosis and “isolated” lesions of the anterior cruciate ligament. Evaluation of the clinical diagnosis. Unfallchirurg. 88:419–423.
13. Wong JWK, Chien EP, Yip DKH, Tang WM, Chin ACW, Peh WCG. (1999) Is MRI necessary to confirm an acute ACL rupture? Hong Kong J Sport Med Sport Sci. 8:1-5.
14. Yoon YS, Rah JH, Park HJ. (1997) A prospective study of the accuracy of clinical examination evaluated by arthroscopy of the knee. Int Orthop. 21: 223–227.
