# Supplementary material for: The diagnostic accuracy of clinical tests for anterior cruciate ligament tears are comparable but the Lachman test has been previously overestimated: a systematic review and meta-analysis
Source: Knee Surg Sports Traumatol Arthrosc. 2022 Feb 12;30(10):3287–303. doi: 10.1007/s00167-022-06898-4 (PMC9464183; doi:10.1007/s00167-022-06898-4)
Supplement: Supplementary file 3 — Supplementary file3 (DOCX 13 KB) [file 167_2022_6898_MOESM3_ESM.docx]

**Supplemental Table 1 Search terms used in each bibliographic database** A search was conducted for relevant studies without restriction on date of publication using the bibliographic databases PubMed, Scopus, MEDLINE and Web of Science. The search terms used were formulated according to the aims of the study, PICOS design and those adapted by literature reviews reporting similar data.Search terms were adjusted according to the bibliographic database to accommodate for differences within each system.

| **Database** | **Search terms** |
| --- | --- |
| PubMed | "ACL" OR "anterior cruciate ligament") AND ("anterior drawer" OR "anterior drawer test" OR "drawer test" OR "pivot" OR "pivot test" OR "pivot shift" OR "pivot shift test" OR "lateral pivot shift" OR "lateral pivot shift test" OR "Lachman" OR "Lachman test" OR "Lachman's test" OR "Lever" OR "Lever test" OR "Lever sign test" OR "Lelli" OR "Lelli test" OR "Lelli's test" OR "MRI" OR "magnetic resonance imaging" |
| Scopus and MEDLINE | ("ACL" OR "anterior cruciate ligament") AND ("anterior drawer" OR "anterior drawer test" OR "drawer test" OR "pivot" OR "pivot test" OR "pivot shift" OR "pivot shift test" OR "lateral pivot shift" OR "lateral pivot shift test" OR "Lachman" OR "Lachman test" OR "Lachman's test" OR "Lever" OR "Lever test" OR "Lever sign test" OR "Lelli" OR "Lelli test" OR "Lelli's test" OR "MRI" OR "magnetic resonance imaging") |
| Web of Science | (ALL=("ACL" OR "anterior cruciate ligament") AND ALL=("anterior drawer" OR "anterior drawer test" OR "drawer test" OR "pivot" OR "pivot test" OR "pivot shift" OR "pivot shift test" OR "lateral pivot shift" OR "lateral pivot shift test" OR "Lachman" OR "Lachman test" OR "Lachman's test" OR "Lever" OR "Lever test" OR "Lever sign test" OR "Lelli" OR "Lelli test" OR "Lelli's test" OR "MRI" OR "magnetic resonance imaging")) |
