# Supplementary material for: The diagnostic accuracy of clinical tests for anterior cruciate ligament tears are comparable but the Lachman test has been previously overestimated: a systematic review and meta-analysis
Source: Knee Surg Sports Traumatol Arthrosc. 2022 Feb 12;30(10):3287–303. doi: 10.1007/s00167-022-06898-4 (PMC9464183; doi:10.1007/s00167-022-06898-4)
Supplement: Supplementary file 4 — Supplementary file4 (DOCX 14 KB) [file 167_2022_6898_MOESM4_ESM.docx]

**Supplemental Table 2 Inclusion and exclusion criteria applied to studies** Inclusion and exclusion criteria were defined for the selection of eligible studies within the review. Studies were deemed eligible if they were randomised controlled trials (RCTs), prospective or retrospective cohort studies evaluating the diagnostic accuracy of the anterior drawer, Lachman, Lever sign and/or pivot shift tests performed according to the original test description in patients without anaesthesia, using magnetic resonance imaging (MRI) and/or arthroscopy as the reference standard. Studies were included irrespective of year of publication or quality of the methods but limited to those with human participants and where English language texts were available in-full.

| **Inclusion criteria** | **Exclusion criteria** |
| --- | --- |
| Randomised controlled trials, prospective and retrospective cohort studies | ACL-injured cohort containing less than five subjects |
| Evaluation of at least one of the clinical tests: the anterior drawer, pivot shift, Lachman or lever sign tests | Multi-ligament knee injuries |
| MRI or Arthroscopy as reference standard | History of previous ACL reconstruction |
| Isolated ACL tear (complete or partial) with or without concomitant meniscal injury | Bucket handle meniscal tears |
| Adult and/or paediatric population – analysed separately | Modification of a test (e.g. prone Lachman test or use of arthrometer) |
| Sensitivity and/or specificity reported | Combined test results |
| Each test performed according to the original test description [24,25,37,42,46,73,75,76] | Equivocal test results |
| Examination without anaesthesia | Examination under anaesthesia |
| English language | Animal or cadaveric studies |
|  | Reviews, systematic reviews, case reports, abstracts, conference proceedings |
