# Supplementary material for: The diagnostic accuracy of clinical tests for anterior cruciate ligament tears are comparable but the Lachman test has been previously overestimated: a systematic review and meta-analysis
Source: Knee Surg Sports Traumatol Arthrosc. 2022 Feb 12;30(10):3287–303. doi: 10.1007/s00167-022-06898-4 (PMC9464183; doi:10.1007/s00167-022-06898-4)
Supplement: Supplementary file 5 — Supplementary file5 (DOCX 14 KB) [file 167_2022_6898_MOESM5_ESM.docx]

**Supplemental Table 3 QUADAS-2 signalling questions for each of the categories**. A standardised assessment of the quality of reporting by primary diagnostic accuracy studies was conducted using QUADAS-2 tool [78]. Upon data extraction, each article was critically appraised against four key domains affecting quality of reported data: patient selection, index test, reference standard, and ‘flow and timing’. Reporting of the onset of injury, concurrent injuries, as well as description of the index test were considered particularly important for this review. Risk of bias and concerns regarding applicability of results were determined with the use of signalling questions adapted by QUADAS (<http://www.bristol.ac.uk/population-health-sciences/projects/quadas/quadas-2/>). Some questions were omitted or adjusted to be relevant to addressing the research aims of this current review.

| **Domain** | **Signalling question** |
| --- | --- |
| Patient selection | Was a consecutive or random sample of patients enrolled? |
|  | Was a case-control design avoided? |
|  | Did the study avoid inappropriate exclusions? |
|  | Overall: Could the selection of patients have introduced bias?  RISK: Low/High/Unclear |
| Patient selection applicability concerns | Is there a concern that the included patients do not match the review question?  CONCERN: Low/High/Unclear |
| Index test | Were the index test results interpreted without knowledge of patient’s clinical history or the results of the reference standard? |
|  | Were the tests performed in a conventional way? |
|  | If multiple tests were used, was the order randomised? |
|  | Overall: Could the conduct of the index test have introduced bias?  RISK: Low/High/Unclear |
| Index test applicability concerns | Is there a concern that the index test or its conduct differ from the review question?  CONCERN: Low/High/Unclear |
| Reference standard | Is the reference standard likely to correctly classify the target condition? |
|  | Were the reference standard results interpreted without knowledge of the results of the index test? |
|  | Overall: Could the reference standard, its conduct or its interpretation have introduced bias?  RISK: Low/High/Unclear |
| Reference standard applicability concerns | Is there a concern that the target condition as defined by the reference standard does not match the review question?  CONCERN: Low/High/Unclear |
| Flow and timing | Was there an appropriate interval between index test(s) and reference standard? |
|  | Did all patients receive a reference standard? |
|  | Did patients receive the same reference standard? |
|  | Were all patients included in the analysis? |
|  | Overall: Could the patient flow have introduced bias?  RISK: Low/High/Unclear |
