# Supplementary material for: The diagnostic accuracy of clinical tests for anterior cruciate ligament tears are comparable but the Lachman test has been previously overestimated: a systematic review and meta-analysis
Source: Knee Surg Sports Traumatol Arthrosc. 2022 Feb 12;30(10):3287–303. doi: 10.1007/s00167-022-06898-4 (PMC9464183; doi:10.1007/s00167-022-06898-4)
Supplement: Supplementary file 6 — Supplementary file6 (DOCX 14 KB) [file 167_2022_6898_MOESM6_ESM.docx]

**Supplemental Table 4: Univariate and bivariate analysis of diagnostic clinical tests separated by arthroscopy or MRI as the reference standard.** Comparison of diagnostic clinical tests (anterior drawer, Lachman, Lever sign and pivot shift) in complete and partial ACL tears, acute and post-acute clinical presentations when univariate and bivariate modelling was performed on data that was separated by arthroscopy or MRI as the reference standard. AUC: area under the curve, BA: bivariate analysis, CI: confidence interval, LR-: negative likelihood ratio, LR+: positive likelihood ratio, Sn: sensitivity, Sp: specificity, UA: univariate analysis.

|  | **Arthroscopy only [95% CI]** | | | | | **MRI only [95% CI]** | | | | |
| --- | --- | --- | --- | --- | --- | --- | --- | --- | --- | --- |
|  | **Sn** | **Sp** | **LR+** | **LR-** | **AUC** | **Sn** | **Sp** | **LR+** | **LR-** | **AUC** |
| **Anterior Drawer (BA)** | 0.85 [0.69; 0.94] | 0.96 [0.86; 0.99] | 25.10 [5.33; 76.10] | 0.17 [0.06; 0.34] | 0.965 | 0.82 [0.75; 0.88] | 0.65 [0.53; 0.75] | 2.37 [1.70-3.32] | 0.28 [0.18-0.42] | 0.821 |
| **Lachman (UA)** | 0.87 [0.79; 0.92] | 0.95 [0.74; 0.99] | 2.79 [1.82; 4.28] | 0.27 [0.19; 0.38] | - | 0.81 [0.57; 0.93] | 0.87 [0.56; 0.97] | 2.65 [1.56; 4.53] | 0.27 [0.15; 0.49] | - |
| **Lachman (BA)** | 0.79 [0.67; 0.87] | 0.88 [0.71; 0.95] | 7.35 [2.54; 17.80] | 0.25 [0.14; 0.42] | 0.884 | 0.84 [0.77; 0.90] | 0.79 [0.61; 0.90] | 4.37 [2.04; 8.85] | 0.21 [0.12; 0.35] | 0.875 |
| **Lever Sign (BA)** | 0.86 [0.70; 0.94] | 0.89 [0.76; 0.95] | 8.44 [3.45; 18.20] | 0.17 [0.06; 0.35] | 0.934 | 0.83 [0.58; 0.95] | 0.93 [0.83; 0.97] | 13.50 [4.97; 30.60] | 0.20 [0.06; 0.45] | 0.952 |
| **Pivot Shift (BA)** | 0.53 [0.43; 0.63] | 0.96 [0.92; 0.98] | 17.2 [6.76; 36.90] | 0.49 [0.38; 0.60] | 0.959 | 0.58 [0.45; 0.71] | 0.91 [0.79; 0.97] | 7.48 [2.87; 16.80] | 0.46 [0.33; 0.61] | 0.855 |
