# Supplementary material for: The diagnostic accuracy of clinical tests for anterior cruciate ligament tears are comparable but the Lachman test has been previously overestimated: a systematic review and meta-analysis
Source: Knee Surg Sports Traumatol Arthrosc. 2022 Feb 12;30(10):3287–303. doi: 10.1007/s00167-022-06898-4 (PMC9464183; doi:10.1007/s00167-022-06898-4)
Supplement: Supplementary file 7 — Supplementary file7 (DOCX 14 KB) [file 167_2022_6898_MOESM7_ESM.docx]

**Supplemental Table 5: Univariate and bivariate analysis of diagnostic clinical tests stratified by complete or partial ACL tear.** Comparison of diagnostic clinical tests (anterior drawer, Lachman, Lever sign and pivot shift) in complete and partial ACL tears, acute and post-acute clinical presentations when univariate and bivariate modelling was performed on data that was stratified by complete or partial tear with arthroscopy and MRI as the reference standard. AUC: area under the curve, BA: bivariate analysis, CI: confidence interval, LR-: negative likelihood ratio, LR+: positive likelihood ratio, Sn: sensitivity, Sp: specificity, UA: univariate analysis.

|  | **Complete tears only [95% CI]** | | | | | **Partial tears only [95% CI]** | | | | |
| --- | --- | --- | --- | --- | --- | --- | --- | --- | --- | --- |
|  | **Sn** | **Sp** | **LR+** | **LR-** | **AUC** | **Sn** | **Sp** | **LR+** | **LR-** | **AUC** |
| **Anterior Drawer (UA)** | 0.76 [0.38; 0.94] | 0.55 [0.41; 0.69] | 1.76 [1.26; 2.46] | 0.44 [0.19; 1.01] | - | 0.58 [0.20; 0.89] | - | 0.99 [0.25; 3.99] | 0.711 [0.18; 2.89] | - |
| **Lachman (UA)** | 0.91 [0.77; 0.97] | 0.84 [0.50; 0.96] | 2.29 [1.57; 3.33] | 0.37 [0.27; 0.53] | - | 0.42 [0.35; 0.49] | - | 0.98 [0.31; 3.08] | 0.96 [0.30; 3.12] | - |
| **Lachman (BA)** | 0.68 [0.54; 0.79] | 0.79 [0.51; 0.93] | 3.73 [1.45; 9.12] | 0.43 [0.29; 0.62] | 0.75 | - | - | - | - | - |
| **Lever Sign (UA)** | 1.00 [0.00; 1.00] | - | 1.97 [0.49; 7.88] | 0.03 [0.005; 0.18] | - | 1.00 [0.00; 1.00] | - | 1.97 [0.49; 7.88] | 0.03 [0.005; 0.18] | - |
| **Pivot Shift (UA)** | 0.48 [0.22; 0.75] | 0.96 [0.90; 0.98] | 1.18 [0.39; 3.54] | 0.67 [0.40; 1.11] | - | 0.44 [0.12; 0.82] | - | 0.75 [0.24; 2.38] | 0.96 [0.29; 3.20] | - |
