# Supplementary material for: The diagnostic accuracy of clinical tests for anterior cruciate ligament tears are comparable but the Lachman test has been previously overestimated: a systematic review and meta-analysis
Source: Knee Surg Sports Traumatol Arthrosc. 2022 Feb 12;30(10):3287–303. doi: 10.1007/s00167-022-06898-4 (PMC9464183; doi:10.1007/s00167-022-06898-4)
Supplement: Supplementary file 8 — Supplementary file8 (DOCX 14 KB) [file 167_2022_6898_MOESM8_ESM.docx]

**Supplemental Table 6: Univariate and bivariate analysis of diagnostic clinical tests stratified by post-acute or acute classification of injury.** Comparison of diagnostic clinical tests (anterior drawer, Lachman, Lever sign and pivot shift) in complete and partial ACL tears, acute and post-acute clinical presentations when univariate and bivariate modelling was performed on data that was stratified by post-acute or acute classification of injury with arthroscopy and MRI as the reference standard. AUC: area under the curve, BA: bivariate analysis, CI: confidence interval, LR-: negative likelihood ratio, LR+: positive likelihood ratio, Sn: sensitivity, Sp: specificity, UA: univariate analysis.

|  | **Post-acute injuries only [95% CI]** | | | | | **Acute injuries only [95% CI]** | | | | |
| --- | --- | --- | --- | --- | --- | --- | --- | --- | --- | --- |
|  | **Sn** | **Sp** | **LR+** | **LR-** | **AUC** | **Sn** | **Sp** | **LR+** | **LR-** | **AUC** |
| **Anterior Drawer (UA)** | 0.94 [0.36; 1.00] | - | 1.57 [0.51; 4.89] | 0.23 [0.04; 1.42] | - | 0.53 [0.35; 0.70] | 0.69 [0.43; 0.86] | 1.52 [0.91; 2.54] | 0.49 [0.31; 0.76] | - |
| **Lachman (UA)** | 0.82 [0.66; 0.91] | 0.85 [0.53; 0.97] | 2.26 [1.47; 3.47] | 0.40 [0.29; 0.55] | - | 0.89 [0.67; 0.97] | 0.62 [0.38; 0.82] | 1.79 [1.13; 2.84] | 0.33 [0.20; 0.53] | - |
| **Lachman (BA)** | 0.70 [0.57; 0.80] | 0.77 [0.53; 0.91] | 3.31 [1.55; 6.98 | 0.41 [0.27; 0.57] | 0.77 | - | - | - | - | - |
| **Lever Sign (UA)** | 1.00 [0.69; 1.00] | - | 1.94 [0.63; 6.01] | 0.06 [0.01; 0.21] | - | 1.00 [0.64; 1.00] | 0.94 [0.79; 0.99] | 5.13 [2.13; 12.38] | 0.10 [0.04; 0.27] | - |
| **Pivot Shift (UA)** | 0.86 [0.56; 0.97] | 0.96 [0.90; 0.98] | 2.64 [1.04; 6.69] | 0.30 [0.11; 0.85] | - | 0.26 [0.14; 0.44] | 0.94 [0.66; 0.99] | 0.66 [0.28; 1.54] | 0.61 [0.47; 0.80] | - |
